# Finite element analysis of femoral neck system in the treatment of Pauwels type III femoral neck fracture

**DOI:** 10.1097/MD.0000000000029450

**Published:** 2022-07-15

**Authors:** Yanbin Teng, Yan Zhang, Chaowei Guo

**Affiliations:** a Yanbin Teng, Department of Orthopedics and Trauma Weifang People’s Hospital, Shandong province, People’s Republic of China, Yan Zhang, Department of Nephrology, Weifang People’s Hospital, Shandong province, People’s Republic of China.; b Department of Orthopedic Surgery, Luoyang Orthopedic Hospital of Henan Province, Henan Province, People’s Republic of China.

**Keywords:** cannulated screw, femoral neck system, finite element analysis, medial buttress plate, Pauwels type III femoral neck fracture

## Abstract

The optimal treatment strategy for femoral neck fractures remained controversial, especially the Pauwels type III femoral neck fracture of young patients was a challenge. Femoral neck system (FNS) was a newly developed internal fixation for treating femoral neck fracture and this study aimed to compare the biomechanical advantages and disadvantages between FNS and 3 cannulated configuration screws (CCS) with or without an additional medial buttress plate (MBP).

In this study, Pauwels type III femoral neck fracture model with an angle of 70° was constructed and 3 different fixation models, FNS, CCS + MBP, CCS alone, were developed. A vertical force of 2100N was applied on the femoral head, then the maximum von Mises stress of whole model, distal femur, femoral head, and internal fixation was recorded, as well as the stress distribution of whole model, proximal fracture section, and internal fixation of the 3 models. Moreover, the maximum displacement of the whole model, distal femur, femoral head, internal fixation, and the relative displacement of the proximal and distal portion was also compared.

The maximum von Mises stress value was 318.302 MPa in FNS, 485.226 MPa in CCS + 1/3 plate, and 425.889 MPa in CCS. The FNS showed lowest maximum von Mises stress values in distal part, femoral head, and internal implant. All fixation configurations were observed stress concentrated at the posteroinferior area of cross-section of femoral head and at the fracture section area of implant; however, FNS had more uniform stress distribution. For displacement, the maximum displacement value was 8.5446 mm in FNS, 8.2863 mm in CCS + 1/3 plate, and 8.3590 mm in CCS. However, FNS had higher maximum displacement in femoral head and internal implant, but lower maximum displacement in the distal part of fracture model. The FNS represented a significantly higher relative displacement between the femoral head and distal femur when compared with the other 2 fixation configurations.

The newly developed FNS could achieve the dual effect of angular stability and sliding compression for the treatment of Pauwels type III femoral neck fractures, which provided superior biomechanical stability than CCS alone and CCS with additional MBP.

## 1. Introduction

Femoral neck fractures are common hip fractures, accounting for approximately half of hip fractures.^[1]^ In younger population, the fracture usually result sfrom high-energy injuries, which tend to extend in a vertically oriented fracture pattern.^[2–4]^ A greater verticality fracture pattern incurs with higher vertical shear stress on the fracture plane and internal plants, which is more likely to lead to internal plant failure and fracture displacement.^[1,3]^ Especially, the incidence of femoral neck shorting,^[5,6]^ nonunion, and femoral head necrosis was higher in Pauwels type III unstable fracture.^[7]^

Inverted triangle configuration of 3 paralleled cannulated lag screws are the most frequently used fixation strategy in vertical as well as other types of femoral neck fractures^[8,9]^; however, this procedure has a high risk of postoperative internal fixation failure, femoral neck shortening, varus deformity, femoral head necrosis, and nonunion.^[5,7]^ To resist the shear forces of vertical fracture and femoral neck shorting, some scholars applied a buttress plate (a contoured 1/3 tubular plate or 3.5-mm locking plate), anteroinferiorly over the fracture line, which had higher fixation stability than the use of 3 cannulated screws alone and lower incidence of nonunion, femoral head necrosis by converting shear forces into compressive forces.^[3,4,10,11]^ The newly applied device, femoral neck system (FNS), was a minimally invasive, antirotation, and sliding compression implants, which had been reported to be more stable than 3 lag screws and DHS (dynamic hip screw) when fixing Pauwels type III femoral neck fractures.^[12,13]^ However, no study has compared the biomechanical properties between the MBP and FNS device, and the ideal fixation device remains undefined. Given this, the aim of this finite element analysis (FEA) study was to compare the biomechanical advantages and disadvantages for treating Pauwels type III femoral neck fractures, as well as provide a theoretical reference for the treatment of such fractures.

## 2. Materials and Methods

### 2.1. Building the Pauwels III femoral neck fracture model

**Figure 1.**
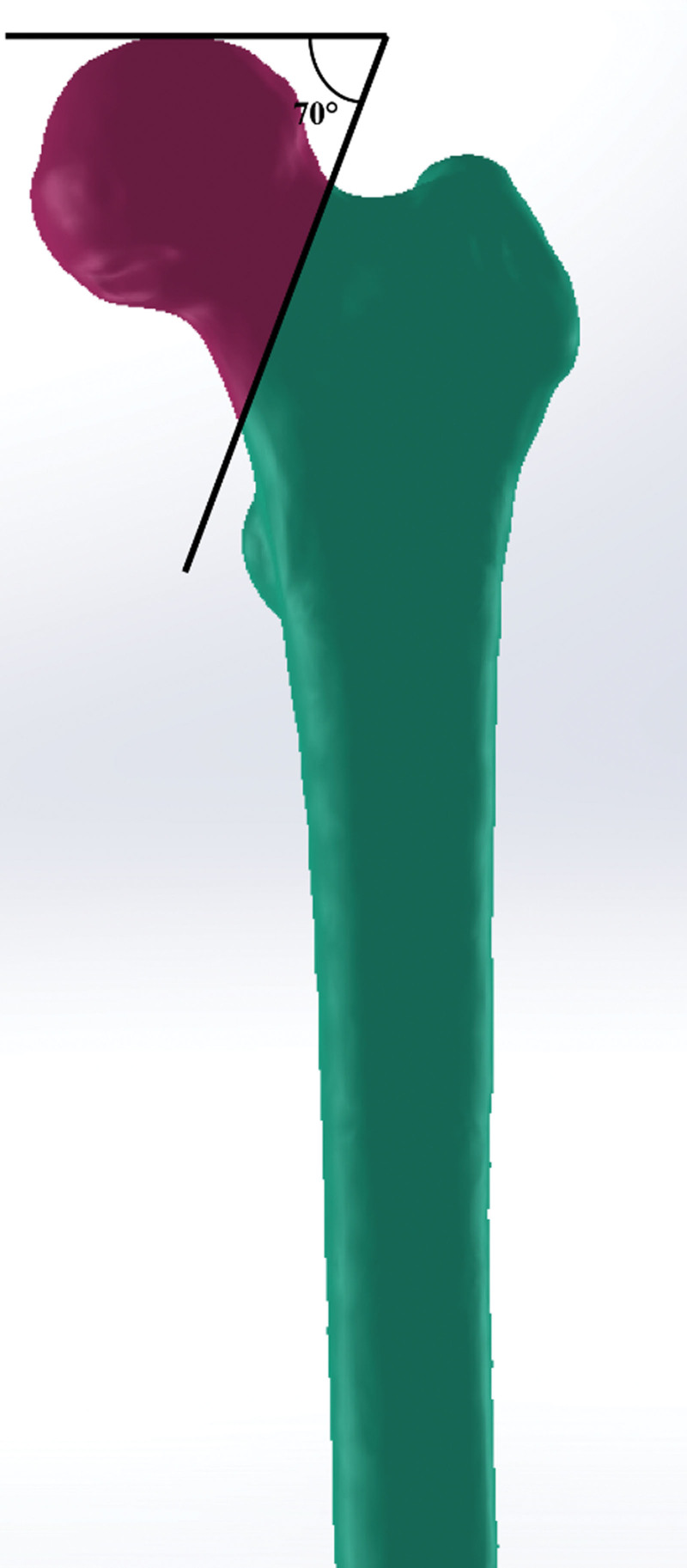
Pauwels type III femoral neck fracture model.

A 22-year-old female patient who signed an informed consent was selected to obtain the 64-slice CT scanned images. Then, the files of DICOM (digital imaging and communications in medicine) format were imported into Mimics 17.0 (Materialise, Belgium) to calculate the 3D model. Then, we created a cutting plate that was across the center of the femoral neck at an angle of 70° with respect to the horizontal plane to simulate a Pauwels type III unstable fracture after the 3D model was smoothed in the 3-matic 12.0 (Materialise, Belgian; Fig. 1.).^[14]^

### 2.2. Building and assembling the different internal fixation model

**Figure 2.**
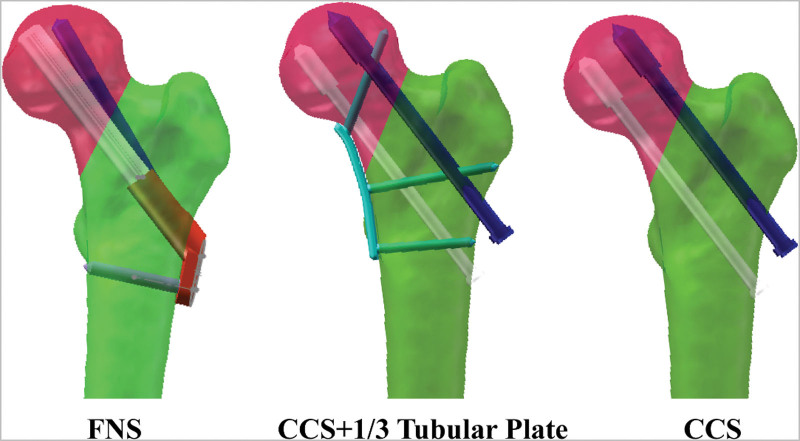
3D models of 3 internal fixations of femoral neck fracture. CCS = cannulated configuration screw, FNS = femoral neck system.

According to the parameters of clinical fixation implants and the guidance of a senior surgeon, 3 kinds of internal fixation models, FNS, inverted triangle of cannulated screws (CCS), and CCS + 1/3 tubular plate, were established and assembled in 3-matic 12.0 (Materialise, Belgian; Fig. 2.). To improve the convergence of FEA model and reduce the stress concentration at small corner, the FNS was separated into 2 parts: an angle of 130° locking plate with a 5.0-mm locking screw at the distal end and a sliding screw with a diameter of 10-mm and a 6.4-mm screw placed at an angle of 7.5° at the proximal end.^[13]^ Similarly, the threaded screw sections of cannulated screws were simplified as a diameter of 7.3-mm and 16-mm long cylinder (shaft diameter, 4.8 mm; cannulated tunnel diameter, 2.8 mm). A 2.7-mm thickness 5-hole 1/3 tubular plate and 3 3.5-mm locking screws were fabricated with 3-matic 12.0 (Materialise, Belgian). Subsequently, each assembled model was meshed by C3D4 tetrahedral elements and checked the mesh quality in the Hypermesh 14.0 (Altair, USA). Then all components were exported as *.inp for assigning material properties.

### 2.3. Material parameters

The aforementioned dataset of *.inp was returned to Mimics for material assignment. Ten graded materials were assigned based on CT grayscale for bone as revealed, and the material properties were assumed to be linear elastic, isotropic, and homogeneous, according to the experience materials assigned formula recommended by the Mimics software (Formula 1). The implants were assigned as Ti-6Al-4V alloy (density: 4.51 g/cm^3^; Young’s modulus: 110 GPa, Poisson’s ratio: 0.3).^[15]^ Then all the models were exported as *.inp again and imported into Abaqus 6-14 (Simulia, France).

Density = 131 + 1.067 × HU (g/cm^3^);

E modulus = 0.004 × density^2.01^ (MPa); (Formula 1)

Poisson’s ratio = 0.3.

### 2.4. Contact settings, boundary conditions, and loading force settings

**Figure 3.**
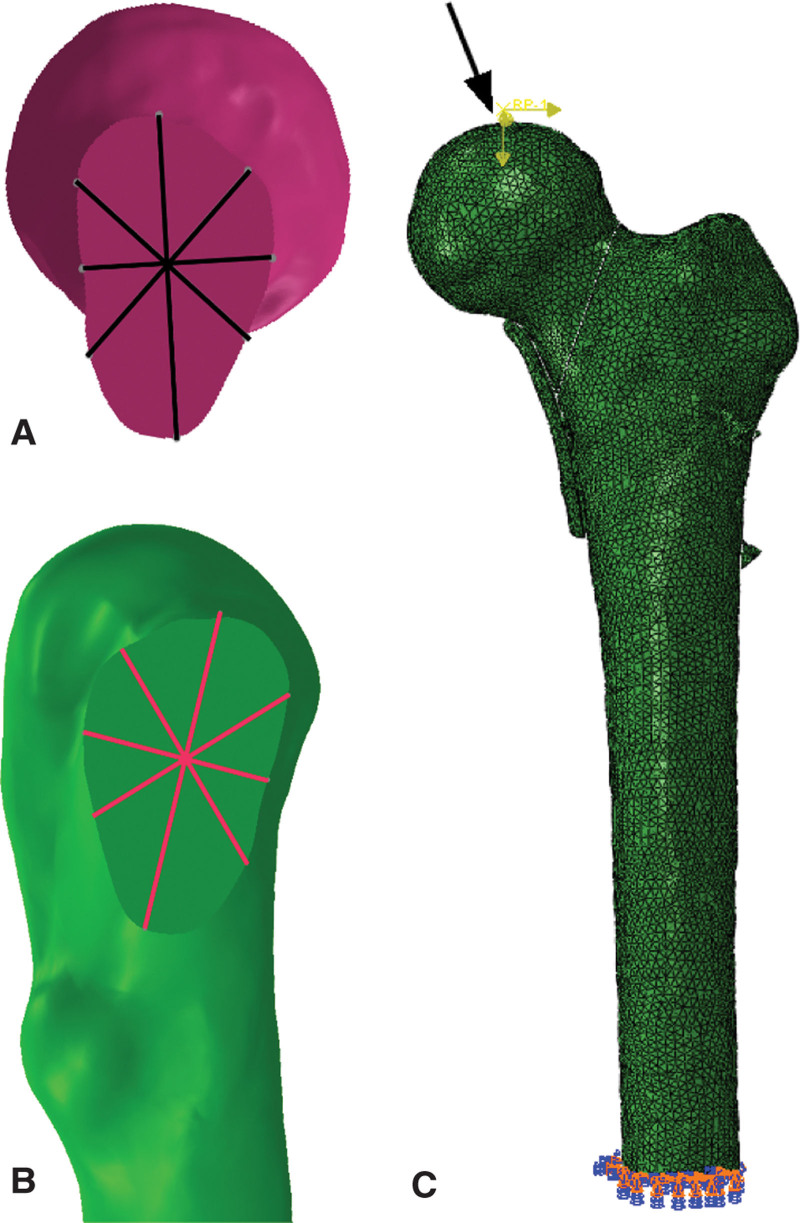
(A) The 8 points defined in proximal fracture surface. (B) The 8 points defined in distal fracture surface. (C) The black arrow is the direction of the force, and the distal area is fixed.

Referring to previous studies, the fracture surface, the contact surface between all screw shaft and bone, and the contact surface between sliding screw and locking plate in FNS were set to friction, with a friction coefficient of 0.46, 0.30, 0.30, respectively. The screw thread–bone interface in all cases was assumed to be fixed. The degrees of freedom of all nodes at the distal end of the femur were set to 0, that is, the displacement of each node at the X, Y, and Z axes was 0. A 3 times body weight downward vertical force (2100 N) was loaded on the femoral head, with lateral tilted 12° and tilted backward 10°, which was based on the anteversion angle of this femur (Fig. 3C).^[16]^

### 2.5. Evaluation criteria

First, the peak von Mises stress (VMS) of whole model, distal femur, femoral head, and internal fixation was recorded, as well as the stress distribution of whole model, proximal fracture section, and internal fixation of the 3 models was observed. Then, except for the maximum displacement of the whole model, distal femur, femoral head, and internal fixation, we defined 8 points with the same coordinates in the proximal and distal fracture surface to record and compare the difference of relative displacement of the 3 models, which was calculated by a difference between the proximal and distal points (Fig. 3A, B).

## 3. Results

**Figure 4.**
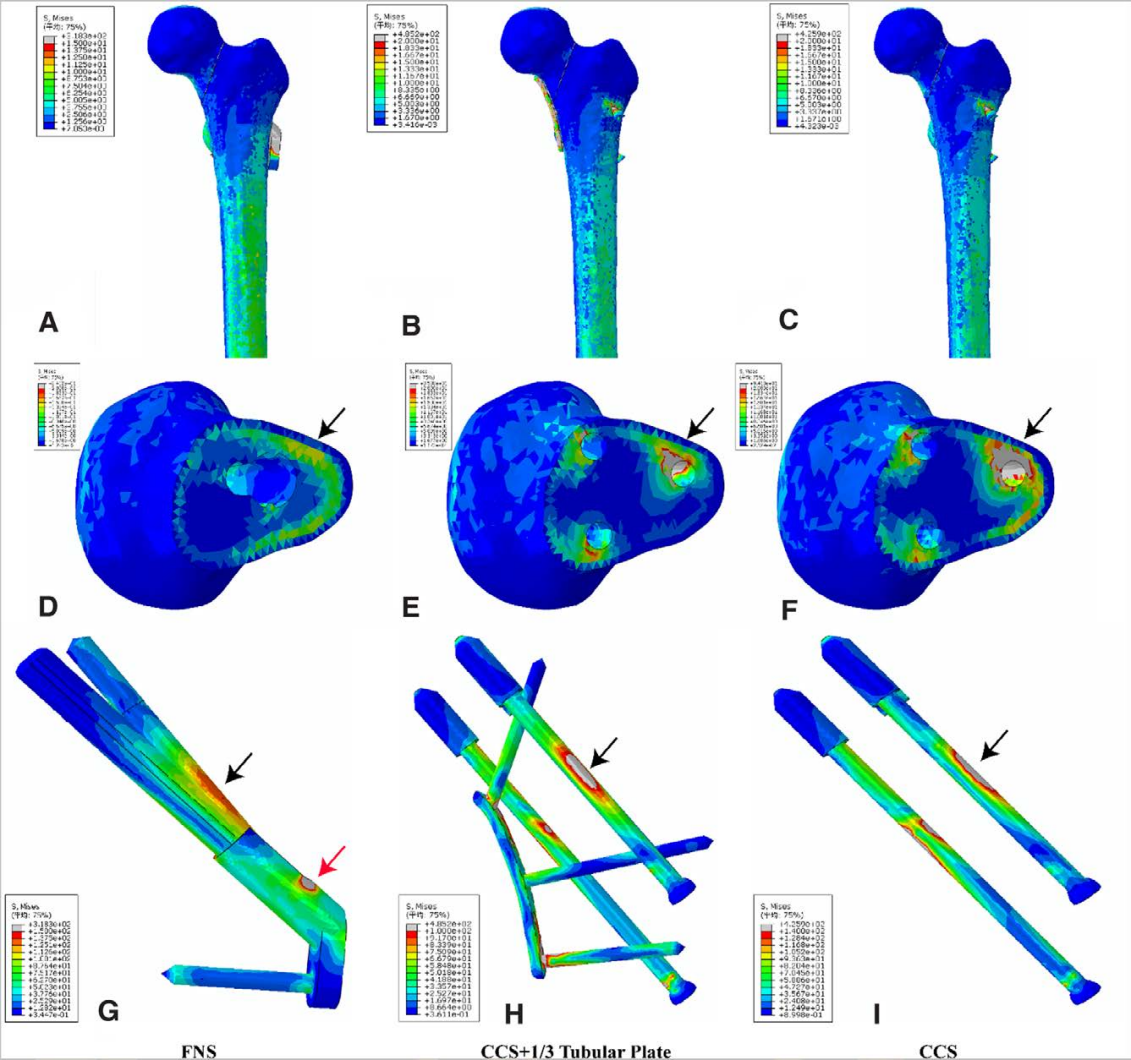
The stress distribution modes. (A–C) The stress distribution of whole model. (D, E) The stress distribution of cross-section of femoral head (noted the stress concentration area was close to the femoral calcar [black arrows]). (G–I) The stress distribution of internal fixation (noted the stress concentration area in the fracture section [black arrows] and the junction area [red arrows]). CCS = cannulated configuration screw, FNS = femoral neck system.

The stress distributions, peak VMS, maximum displacement, and relative displacement are shown in Figure 4 and Table 1.

**Table 1:** Analysis results.

	Parameter	FNS	CCS + 1/3 plate	CCS
Peak VMS (Mpa)	Whole model	318.302	485.226	425.889
	Distal femur	117.772	136.525	154.749
	Femoral head	24.024	85.882	94.179
	Internal fixation	318.302	485.226	425.889
Maximum displacement (mm)	Whole model	8.5446	8.2863	8.3590
	Distal femur	6.2919	6.3372	6.3491
	Femoral head	8.3493	8.1088	8.1825
	Internal fixation	8.0402	7.8986	7.9673
Relative displacement (mm)		0.2115 ± 0.2677	0.0585 ± 0.0434	0.0807 ± 0.0435

CCS = cannulated configuration screw, FNS = femoral neck system, VMS = Von Mises stress.

### 3.1. Von Mises stress

The differences in VMS distribution for the 3 groups are documented as Figure 4. In the whole model and distal femur, the stresses appeared to be concentrated at the lower part and internal fixation area and whole model peak VMS was 318.302 MPa in FNS, 485.226 MPa in CCS + 1/3 tubular plate, and 425.889 MPa in CCS. Also, the distal femur peak VMS was lower in FNS. In the cross-section of femoral head, the posteroinferior area, which was closest to the femoral calcar, was the stress concentration area in each group (Fig. 4D, E). The peak VMS of each femoral head was 24.024 MPa, 85.882 MPa, and 94.179 MPa in FNS, CCS + 1/3 tubular plate, and CCS, respectively. The stresses of internal fixation appeared to be concentrated at the site closest to fracture section in each group, while more uniform distribution was observed in FNS. The internal fixation VMS of FNS was reduced by 166.924 MPa than CCS + 1/3 tubular plate and 107.587 MPa than CCS, respectively (Fig. 4G–I).

### 3.2. Displacement

The maximum displacement of each model is shown in Table 1. The maximum displacement of whole model was 8.5446, 8.2863, 8.3590 mm in FNS, CCS + 1/3 tubular plate, and CCS, respectively. Interestingly, the maximum displacement of each component occurred at the femoral head, and 8.3493 mm in FNS, 8.1088 mm in CCS + 1/3 tubular plate, and 8.1825 mm in CCS. Relatively low displacement was the distal part, which was 6.29196 mm in FNS, 6.3372 mm in CCS + 1/3 tubular plate, and 6.3491 mm in CCS. As for the internal fixation, the FNS was 1.02 times than CCS + 1/3 tubular plate and 1.01 times than CCS, respectively. FNS group had significant higher mean relative displacement than CCS + 1/3 tubular plate group (*P* = .003) and CCS group (*P* = .012), but no difference between CCS + 1/3 tubular tubular plate group and CCS group (*P* = .66).

## 4. Discussion

The results of this FEA suggested that newly developed internal fixation FNS had lower peak stresses of the whole model, distal femur, femoral head, and internal fixation in treating Pauwels type III femoral neck fractures when compared with the other 2 methods. However, the highest maximum displacement of the 3 fixation strategies, and a significant higher relative displacement between the distal and proximal portion was observed in FNS. Additionally, additional MBP did improve the fixation stability as compared to cannulated screw fixation alone.

Anatomic reduction and stable internal fixation for femoral neck fracture were necessary to achieve satisfactory clinical outcomes^[4]^; however, the optimal fixation option was no consensus. Although cannulated screws were widely used for femoral neck fractures, implant failure, femoral neck shorting, and nonunion often occurred postoperatively.^[5,7]^ The main reason was the sliding compression force, perpendicular to the fracture line, was just 1 component of resultant force of hip, and another component increased the shear stress on the fracture plane and internal plants, which may cause the displacement of the fracture end and fixation failure.^[1,3]^ Moreover, the greater the vertical fracture line, the higher the shear stress, especially the anteroinferior area (Fig. 4C). To resist the shear forces of vertical fracture, some scholars applied a MBP anteroinferiorly over the fracture line, which was proved to be more stable than 3 cannulated screws alone and lower incidence of nonunion, femoral head necrosis, especially in vertical femoral neck fractures.^[3,4,10,11]^ This study also confirmed that the medial buttress plate (MBP) really decreased the stress of cross-section of femoral head as well as the displacement of hip and fractures ends; however, MBP bore more varus stress (Fig. 4H), which may increase the breakage risk of implants. Additionally, the increased stress maybe related to the use of locking plate in this study, which made this configuration a length-stable fixation model, prevented the dynamic compression between the fracture fragments, and may increase the risk of nonunion in actual clinical application. So even though the plate breakage rate was low in clinical application,^[3,4,10,11]^ whether the 2.7-mm plate effectively buttresses the fracture should be further researched. Furthermore, the overall complications rate of MBP was 17.9%, reported by Ye et al,^[4]^ which was favorable and comparable to the previously reported data.^[7]^ Although the application of MBP would not increase the risk of iatrogenic femoral head necrosis,^[4,16]^ some unique complications associated with the placed position of plate should be noticed like hip impingement.^[17]^

FNS was a newly developed implant, combined the angular stability, and sliding compression, which showed superior mechanical property than Hansson Pins,^[12]^ dynamic hip screw, and cannulated screws^[13]^ in vitro experiment. And this study did confirm the lowest VMS (Table 1) and more uniform distribution in FNS fixed model when compared with the other 2 fixation configurations (Fig. 4), which indicated that the FNS fixation may reduce the risk of postoperative internal fixation failure. Which was attributed to the design of “nail in nail”: a 6.4-mm antirotation screw placed at an angle of 7.5° was locked with the 10-mm sliding bolt, then they were combined with an angle of 130° locking plate and fixed with femoral shaft.^[13]^ The implant volume has an important influence on the revascularization of the femoral head. It has been reported that a small implant volume decreases the incidence of femoral head necrosis.^[18]^ According to the diameters of 2 internal fixations, the volume of cannulated screws was larger than that of the FNS group. On the one hand, the locking mechanism of sliding bolt and ARScrew, locking plate, and distal femoral shaft provided primary rotational and angular stability postoperatively. Several studies have found that the revascularization of the femoral head is highly depended on the stability of the femoral neck fractures.^[19,20]^ The stability of implant makes a significant impact on reducing femoral head necrosis and promoting bone healing. Based on our results, the peak VMS in the FNS group was lower than that of the CCS group and CCS + 1/3 tubular plate group. On the other hand, the dynamic compression mechanism between the sliding bolt and locking plate allowed the proximal portion had maximum of 20-mm sliding theoretically, which would make more uniform stress conduction and distribution within the proximal femur and lower VMS in femoral head and implant itself. Moreover, the dynamic compression mechanism could explain that the FNS had highest maximum displacement and significant higher mean relative displacement between the proximal and distal portion in this study, because the contact surface between sliding screw and locking plate in FNS was set as friction. Although there is no literature to support this idea, it is a reasonable setting to simulate the actual working mechanism, and the FEA results was comparable to the cadaveric model biomechanical study.^[12,13]^ In clinical practice, Zhou et al^[20]^ found that FNS has lower VAS scores, higher Haris score, and lower incidence of complications than cannulated screws. However, the intraoperative blood loss was significantly higher in the FNS group. Consistent findings were observed by Tang et al,^[21]^ the FNS is a suitable alternative to cannulated screws for the treatment of unstable femoral neck fractures. Except for this, a stress increased area was observed at the junction area between the sliding blot and locking plate (Fig. 4G; average 371.667 MPa). The reason for this may be the short sliding sleeve and long working distance of blot, which resulted in the stress inevitably becoming concentrated at the junction when a vertical downward force was loaded. Therefore, the increased stress at the junction area needed attention, and perhaps lengthening or reinforcing the stiffness of the sliding sleeve was an improvement in the future. Furthermore, all of the 3 fixation configurations were observed with a stress concentration at the site closest to the fracture section (Fig. 4G–I); nonetheless, the FNS showed lower stress (average 106.185 MPa) value than inverted triangle of cannulate screws alone (average 137.542 MPa) and CCS + 1/3 tubular plate (average 124.791 MPa), which was further confirmed by the superior mechanical properties of FNS. Although the FNS provides a rotator and angular structure and also provides a dynamic sliding system, the clinical efficacy of FNS needs to be further evaluated by more multicentre randomized controlled clinical studies.

### 4.1. Limitation

Although this was the first study to analyze the biomechanical properties of FNS and raise some potential deficiencies of this device, there were still some limitations. First, to improve the convergence of models, the threaded screw sections of all implants were simplified as cylinders and the effects of surrounding muscles and ligaments on fracture were ignored. Second, this study only tested the stability of 2-part fractures and the most vertically oriented fracture pattern (Pauwels type III). Although the results were encouraging, the actual clinical cases were more complex. Moreover, the CT data used came from a healthy teenager, whose bone quality and bone morphology was different from the elderly patients and middle-aged patients. This study was only a preliminary discussion, and further comparisons require a larger sample of clinical research applications.

## 5. Conclusion

The present finite element modeling results revealed that the newly developed FNS could achieve the dual effect of angular stability and sliding compression for treating Pauwels type III femoral neck fractures, which provided superior biomechanical stability than CCS alone and CCS with additional MBP. More pieces of evidence from clinical practice and additional biomechanical study in the management of unstable femoral neck fractures were required to confirm our findings.

## Author contributions

Yanbin Teng and Chaowei Guo provided the idea and finished the experiment. Yan Zhang collected and analyzed the data. All authors read and approved the final manuscript.
